# The Cost of Acute Respiratory Infections With Cough Among Urban Aboriginal and Torres Strait Islander Children

**DOI:** 10.3389/fped.2018.00379

**Published:** 2018-12-03

**Authors:** Yolanda G. Lovie-Toon, Steven M. McPhail, Yin To Au-Yeung, Kerry K. Hall, Anne B. Chang, Dimitrios Vagenas, Michael E. Otim, Kerry-Ann F. O'Grady

**Affiliations:** ^1^Institute of Health and Biomedical Innovation, Queensland University of Technology, South Brisbane, QLD, Australia; ^2^Centre for Functioning and Health Research, Metro South Health, Woolloongabba, QLD, Australia; ^3^School of Human Services and Social Work, Menzies Health Institute Queensland, Griffith University, Nathan, QLD, Australia; ^4^Menzies School of Health Research, Charles Darwin University, Casuarina, NT, Australia; ^5^Lady Cilento Children's Hospital, Queensland Children's Hospital and Health Services, South Brisbane, QLD, Australia; ^6^Department of Health Services Administration, College of Health Sciences, University of Sharjah, Sharjah, United Arab Emirates; ^7^Department of Public Health, Virtual University Uganda, Kampala, Uganda

**Keywords:** respiratory, cough, children, aboriginal, indigenous, cost, economic, Australian

## Abstract

**Introduction:** Acute respiratory infections with cough (ARIwC) contribute considerably to childhood morbidity, yet few studies have examined the cost of these illnesses among Australian children. Moreover, of the few studies that have, none are inclusive of Aboriginal and/or Torres Strait Islander children, despite this population experiencing a greater burden of respiratory illnesses. This study aimed to determine the costs of ARIwC among urban Aboriginal and/or Torres Strait Islander children from the perspective of caretakers, the public healthcare system, and employers.

**Methods:** This cost of illness study used data collected from Aboriginal and/or Torres Strait Islander children aged <5 years enrolled in a 12 month prospective cohort study conducted through an urban primary healthcare clinic in Queensland, Australia. Illness-related resource use was collected for each episode of ARIwC reported, and costed at market rates. Linear regression was used to (a) examine cost per episode by season of illness onset and cough duration and (b) examine cost per month of observation by baseline child and family characteristics.

**Results:** During the study period, a total of 264 episodes of ARIwC were reported among 138 children. The total mean cost was estimated to be $AU252 per non-hospitalized episode (95%CI 169–334). Caretakers, the public healthcare system and employers incurred 44, 39, and 17% of costs per episode, respectively. After accounting for months of completed follow-ups, the total mean cost per child per year was estimated to be $991 (95%CI 514–1468). Winter episodes and episodes resulting in chronic cough were associated with significantly higher costs per episode. A prior history of wheezing, connections to traditional lands and parent/guardian belief that antibiotics should be given until symptoms resolved were associated with significantly higher cost per child month of observation.

**Conclusion:** The cost of ARIwC in this predominantly disadvantaged population is substantial, particularly for caretakers and this needs to be considered in both clinical management and public health initiatives. The importance of cultural factors on health and burden of illness should not be overlooked. Further research into the prevention of chronic cough may play an important role in reducing the economic burden of pediatric respiratory infections.

## Introduction

Acute respiratory infections (ARIs) are leading causes of childhood morbidity worldwide (1). Australian children typically experience between 2 and 7 ARI episodes annually (2–5), the majority of which are managed by families and primary health care services (3, 4). Cough, a common symptom of both acute and chronic respiratory illnesses, consistently remains among the most frequent reasons parents seek health care for their children (6). The symptom of cough often persists longer than other symptoms associated with ARI such as runny nose and fever (7–9) and children with persistent cough report high use of health care services (10). ARIs with cough (ARIwC) are of particular interest as they are more likely to reflect lower airway involvement. This is especially true when the cough is wet and prolonged, which may be associated with an increased risk of chronic lung illnesses in later ages (11).

Despite the burden of childhood ARI to families and health services, there is a paucity of Australian and international studies comprehensively examining the economic burden of ARIs among non-hospitalized cohorts. Three Australian studies have sought to estimate the cost of a non-hospitalized ARI episode among young children (12–14); two of these were conducted over 14 years ago (12, 13). The first study estimated the mean cost per episode to be AU$309 (2001–2002 financial year) (12), the second to be AU$241 (2003) (13), and the third to be AU$626 (2010) (14). While all three studies stated they were conducted from a societal perspective, there are variations in what and how items were costed. The common focus of these studies was on (a) episodes of viral ARIs, particularly influenza and influenza-like-illness (ILI), through the use of specific ILI case-definitions, and (b) on children attending childcare centers and/or urban mainstream child and maternal health services. Consequently, these studies largely exclude bacterial respiratory infections, and disproportionately include non-Indigenous Australian children from high socioeconomic backgrounds.

Among Aboriginal and/or Torres Strait Islander Australian children the burden of respiratory illnesses is considerably greater compared to non-Indigenous Australian children (15). ARIs are the main reason healthcare is sought for Aboriginal and/or Torres Strait Islander children aged ≤ 5 years in both urban (16) and remote (17) communities. Furthermore, parents and carers of Aboriginal and/or Torres Strait Islander children with chronic respiratory illnesses report being worried and concerned about preventing and managing their child's illness given their lack of economic resources (18). To date, no cohort studies have estimated the economic burden of ARIs outside of the hospital setting among Aboriginal and/or Torres Strait Islander children. National data has estimated that hospitalization expenditure for ARIs in 2010–2011 was 3.1 times greater among all Aboriginal and/or Torres Strait Islander Australians than non-Indigenous Australians (AU$184 vs. AU$59 per-person) (19). These values likely underestimate the economic burden, as costs to caretakers and third parties were not included (19). Furthermore, these findings give no indication of expenditure on ARIs outside of the hospital setting.

Thus, this study aimed to determine the costs of ARIwC among urban Aboriginal and/or Torres Strait Islander children from the perspective of caretakers (Box 1), the public healthcare system and employers. The objectives were to (a) estimate the total cost of ARIwC per episode, per child-month of observation, and per child-year (b) examine the distribution of costs incurred between the three sectors, (c) examine the influence of cough duration and season of illness onset on cost per episode, and (d) examine the association between baseline child/family characteristics and cost per month of observation.

Box 1 **Glossary of terms.**

**Table:** 

Bulk-billed/non bulk-billed (20)	In Australia, a healthcare service which is bulk-billed refers to a healthcare service in which the government funds a pre-specified amount for the service with no additional out-of-pocket expenses to the patient as per the Australian Government's Medicare Benefits Scheme (MBS). A non-bulk-billed healthcare service refers to a service in which there is an additional cost above the amount specified by the MBS, which is charged by the healthcare service and must then be met by the patient.
Caretakers	Includes any immediate and extended family, non-biological carers or guardians, and other members of the community involved in the care of the child (parents, step-parents, siblings, grandparents, friends, foster carers, etc.). Does not include people whose professional role is as a “caretaker,” i.e., childcare workers or healthcare workers.
Closing the Gap (CTG) scheme (21)	A scheme in conjunction with the Pharmaceutical Benefits Scheme which further subsidizes medications for Aboriginal and/or Torres Strait Islander patients meaning little or no patient co-payments are required for medicines listed on the Pharmaceutical Benefits Schedule.
Cultural connections	In this study, the term cultural connections was used to refer to use and/or interaction with Aboriginal and/or Torres Strait Islander art, music, dance, storytelling, food, traditional medicine, television, radio, newspaper, and internet sites.
Medicare Benefits Scheme and Schedule (MBS) (22)	The Medicare Benefits Scheme is a national, government-funded scheme that subsidizes the cost of personal medical and allied health services for all Australians. All services included in the scheme are listed on the Medicare Benefits Schedule.
Non-work activities	Includes recreation and leisure activities, usual home duties such as caring for children and housework, usual appointments, studying, volunteering, sleep etc., regardless of whether in paid employment or not.
Pharmaceutical Benefits Scheme and Schedule (PBS) (22)	The Pharmaceutical Benefits Scheme is a national, government-funded scheme that subsidizes the cost of a wide range of pharmaceutical drugs for all Australians. All medicinal products included in the scheme, and their uses, are listed on the Pharmaceutical Benefits Schedule.
Stolen Generation (23)	The Stolen Generation refers to Aboriginal and/or Torres Strait Islander people who, as children, experienced separation from their families and communities by compulsion, duress or undue influence as part of Government laws, policies, and practices at the time.

## Methods

A glossary including terms related to the cost perspective, Australian healthcare system, and Aboriginal and/or Torres Strait Islander peoples and communities, is provided in Box 1.

### Study Design and Population

A 12-month prospective observational cohort study was conducted through a primary healthcare clinic in South-East Queensland, Australia. The protocol for the overarching study has been published (24). Children attending the clinic for any reason were eligible for enrolment if they were (i) aged ≤ 5 years, (ii) a registered patient with the clinic, and (iii) planned to stay within the study area during the 12-month study period. Written informed consent was obtained from a parent/guardian prior to enrolment. Recruitment occurred between February 2013 and November 2015; data collection concluded in November 2016. This present study includes data from all Aboriginal and/or Torres Strait Islander children enrolled in the broader cohort who were not diagnosed with any chronic respiratory disease (excluding asthma) by a respiratory physician during the study period. In accordance with Australian national standards, Australian Indigenous status is a self-identification measure determined through self-report (25).

The study was approved by the ethics committees of the Queensland Children's Health Services (HREC/12/QRCH/169), University of Queensland (2012001395) and Queensland University of Technology (1300000741). The study was registered with the Australia New Zealand Clinical Trials Registry (ACTRN12614001214628). Cultural oversight was provided by an Indigenous Research Reference Group.

### Data Collection

**Figure 1:**
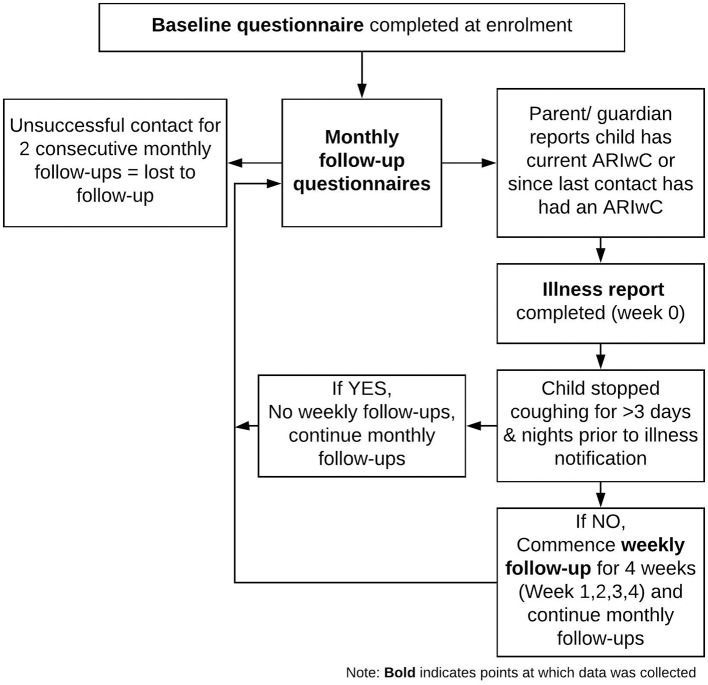
Study procedures.

Figure 1 shows the study procedures and timepoints of data collection. Baseline questionnaires detailing demographics, household characteristics, child and family health behaviors, and medical history, were completed at enrolment. Follow-up questionnaires were administered on a monthly basis via phone, or in person if the parent/guardian was attending the health clinic at a scheduled follow-up timepoint. These questionnaires asked about the health of the child, and health-related resource use for any reason (including visits to a general practice, emergency department, dentist or other health professionals; hospitalizations; and medications and immunizations), in the past month. When contact was unsuccessful for two consecutive months, participants were considered lost to follow-up.

If, at any point throughout the study, the child had an ARIwC (defined as any acute illness with cough), research staff completed an “illness report.” Illness reports collected all resource use related to the ARIwC since illness onset. If, at the time of illness notification the ARIwC episode had resolved (resolution defined as no cough for at least 3 days and nights), monthly follow-ups continued as scheduled. If, at the time of illness notification, the ARIwC episode was ongoing, weekly follow-ups commenced for 4 weeks. Weekly follow-ups collected information on cough characteristics, all resource use related to the ARIwC, and parents/guardians concerns about money spent on the child's ARIwC illness, in the past week. See Supplementary Table 1 for questionnaires used for illness reports and weekly follow-ups. Concerns about money spent on the child's ARIwC illness was measured using a 7-point Likert type scale ranging from “very, very worried/concerned” to “not worried/concerned.” All the resource use data collected throughout the study were parent/guardian-reported.

### Costing

Illness-related costs were evaluated from the perspective of caretakers, the public healthcare system, and employers. With the exception of childcare fees which were directly reported by parents/guardians, a standard unit cost was obtained from published external sources and applied to each item of resource use in Australian Dollars at 2017 prices. Table 1 presents the items of resource use costed for each sector, the unit cost for each item, and the source of that cost information.

**Table 1:** Unit costs in Australian dollars 2017.

**Item**	**Unit cost**	**Source**	
**SECTOR: CARETAKERS**			
Non bulk billed healthcare services^‡^		Australian Medical Association (26)	Medicare Benefits Schedule (27)
General practice (GP) visit	$41.95	Item no. AA020	Item no. 23, page 141
After hours GP home visit	$101.05	Item no. AA140	Item no. 5023, page 212
Specialist visit	$201.70	Item no. AJ010	Item no. 110, page 151
PBS medications	*Various depending on the medication*	Schedule of Pharmaceutical Benefits (28)	
Over-the-counter medications	*Various depending on the medication*	Chemist Warehouse online Pharmacy (29)	
Time off work with unpaid leave^§^	Males: $43.76/h Females: $37.08/h	Australian Bureau of Statistics (30)	
Time off non-work activities^§^^#^	All persons: $41.26/h	Australian Bureau of Statistics (30)	
Childcare fees already paid	N/A	Self-reported by person completing questionnaire	
**SECTOR: PUBLIC HEALTHCARE SYSTEM**			
General practice visit (bulk billed or non-bulk billed)	$37.05	Medicare Benefits Schedule, item no. 23, page 141 (27)	
After hours home visit by general practitioner (bulk billed or non-bulk billed)	$74.95	Medicare Benefits Schedule, item no. 5023, page 212 (27)	
Specialist visit (bulk billed or non-bulk billed)	$128.30	Medicare Benefits Schedule, item no. 110, page 151 (27)	
Non-admitted emergency department presentation, triage category unknown	$489.90^¥^	National Hospital Cost Data Collection (31)	
Public hospitalization, diagnosis of:		National Hospital Cost Data Collection (32)	
Influenza/viral infection/LRTI	$4523.51^¥^	DRG E62B	
Bronchitis	$2626.92^¥^	DRG E69B	
Bronchiolitis	$3860.32^¥^	DRG E70B	
Diagnostic tests		Medicare Benefits Schedule (27)	
Chest x-ray	$20.10	Item no. 58505, page 889	
Full blood count	$14.45	Item no. 65070, page 1023	
Radioallergosorbet test	$25.75	Item no. 71137, page 1058	
Standard biochemistry profile	$15.05	Item no. 66512, page 1029	
Sweat test	$31.65	Item no. 12200, page 272	
Thyroid function test	$29.60	Item no. 66719, page 1037	
PBS Medications	*Various depending on the medication*	Schedule of Pharmaceutical Benefits (28)	
**SECTOR: THIRD PARTIES (EMPLOYERS)**			
Time off work with paid leave^§^	Males: $43.76/h Females: $37.08/h	Australian Bureau of Statistics (30)	

‡ For non-bulk billed healthcare services the cost to caretakers is equal to the AMA fee minus the MBS rebate.

§ *Used ABS reported average weekly earnings, divided by 38 to obtain cost per hour*.

# Data on this item of resource use not collected in the illness reports administered at time of illness notification

Given the triage category of ED presentations was unknown amongst our cohort, the mean cost of an ED presentation was estimated by summing the cost of an ED presentation of triage category 2, 3, 4, and 5 (URG 55, 95, 108, and 115) and dividing by 4.

¥ *Costs have been adjusted for inflation*.

For medications, a cost per dose for each type of medication was calculated and applied to the number of doses received per episode. Unit costs for medications were valued at concession prices given all children enrolled in the study were either on welfare benefits (e.g., unemployment benefits) or participating in the Close the Gap (CTG) scheme (Box 1). Health care visits were costed differently depending on whether the parent/guardian reported that the health care visit was bulk-billed or not (Box 1). Time off work and time off non-work activities were valued equally using the opportunity costing method (33). Using this method, the cost of time forgone (from both work and non-work activities) was estimated using average Australian hourly earnings to represent the potential economic benefit the caregiver missed out on when needing to care for the child, over other activities. All unit costs were either sourced directly in 2017 prices, or were adjusted for inflation from the reported year to 2017 prices using inflation rates reported by the Reserve Bank of Australia (34). When unit costs went over a financial year, e.g., 2014–2015, the later year was used as the base year for adjustment. No discount rate was applied as costs were only examined over a 12-month period.

### Statistical Analyses

Analyses were undertaken using Stata v15 (StataCorp, College Station, TX, USA). Multiple logistic regression models were used to examine characteristics associated with enrolment (compared to non-enrolment), reporting ≥1 ARIwC episode during the study period (compared to reporting no ARIwC episodes) and completion of all weekly follow-ups following illness notification (compared to some or no weekly follow-ups completed).

Costs were calculated for each sector, at each timepoint, and then summed to obtain a total cost per episode. The cost of episodes involving a hospitalization were reported separately and then excluded from further analyses. The total cost per episode was summarized using means and 95% confidence intervals (CIs), and medians and interquartile ranges (IQR), for episodes with complete data on illness-related resource use.

To account for the episodes with incomplete data on illness-related resource use, multiple imputation was used (35, 36). Incomplete data were either a result of parent/guardian non-response to weekly contact attempts, or missed contact attempts by staff. The proportion of weekly follow-ups that were incomplete at each weekly timepoint was ≤ 20%. Approximately 10% of episodes requiring weekly follow-ups completed no follow-ups. Incomplete data were significantly more likely to occur among episodes in children: (i) with no family history of lung disease, (ii) from families who didn't maintain cultural connections at home (Box 1), (iii) who had spent time living in an Aboriginal/Torres Strait community outside Brisbane in the 12 months prior to enrolment, and (iv) exposed to household tobacco smoke (indoors, outdoors and/or whilst in the car).

Sequential imputation using chained equations with linear regression was used to impute values for costs at each timepoint (4 weekly timepoints), for each sector (caretakers, public healthcare system, employers) (37). Variables associated with cost (ARIwC episode number during the study, season of onset, whether medications were taken for their cough between illness onset and illness notification, whether the child was attending childcare at time of enrolment) informed the multiple imputation model. Twenty imputed datasets were generated. The values of the imputed variables were estimated by pooling the results across each imputed dataset, as per Rubin's rules (38). For the imputed dataset, means and 95% CIs were used to summarize costs by timepoint and sector, as well as the total cost per episode. The proportion of costs incurred by each sector per episode was also calculated.

Simple linear regression was used to examine differences in the mean cost per episode by season of illness onset, and by cough duration. Season of illness onset was categorized as spring (September–November), summer (December–February), autumn (March–May), winter (June–August) using date of illness onset. Cough duration was categorized as acute cough (cough lasting <2 weeks), sub-acute (cough lasting 2–4 weeks), chronic cough (cough lasting >4 weeks) (39), or unknown if cough duration was unable to be determined due to missing data.

The total cost of each episode was summed to obtain a total cost per child during the study period. For each child, the total cost reported during the study period was divided by the number of months of follow-up completed, to obtain a cost per child-month of observation. The cost per month of observation was then multiplied by 12 in order to estimate the annual cost of non-hospitalized ARIwC. Simple linear regression was used to examine the association between cost per month of observation and the baseline characteristics presented in Supplementary Table 2. Any characteristics with a *p*-value ≤ 0.1 were entered into a multiple linear regression model. A backwards elimination approach was used to achieve the final model; characteristics with a *p*-value < 0.05 were retained.

## Results

**Figure 2:**
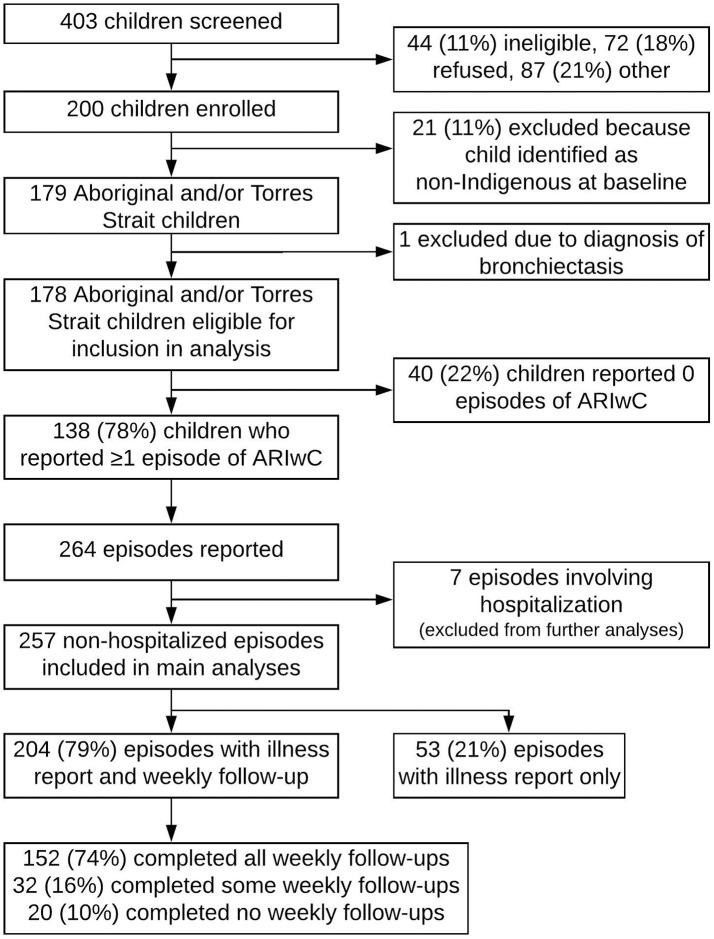
Numbers screened, enrolled, and included in analyses.

Details of the cohort included in our analyses are presented in Figure 2. A total of 200 children were enrolled into the overarching study. There were no significant differences between those enrolled (*n* = 200) and not enrolled (*n* = 203), in respect to sex (*p* = 0.51), however there were differences in age. A significantly greater proportion of children aged 0–2 years were enrolled compared to not enrolled (*p* = 0.02). Children identified as non-Indigenous at baseline (*n* = 21) and children who were diagnosed with a chronic lung disease during the study period (*n* = 1) were excluded from the present study. The median age of the 178 remaining children eligible for inclusion in the present study was 1.5 years (IQR 0.6–2.9); 49% were female, 33% were attending childcare or preschool ≥1 day a week, 90% of primary carer's were receiving government welfare benefits, 5% had private health insurance that covered the child, and 43% of fathers, and 14% of mothers, were employed (Supplementary Table 2). Of these 178 children, 138 reported ≥1 ARIwC episode during the study period and 40 reported no ARIwC episodes. Children who reported ≥1 ARIwC episode had a median of 10 (IQR 8–12) monthly follow-ups completed, compared to a median of 4 (IQR 1–10) for children with no ARIwC episodes reported. In regression models that accounted for months of follow-up, children who had wheezing in the 12 months prior to enrolment were significantly more likely to report experiencing ≥1 ARIwC during the study period (*p* = 0.02), as were children whose primary carer knew definitively that they did or did not have a family member from the Stolen Generation (Box 1; compared to children whose primary carer who did not know; *p* < 0.01).

The 7 episodes that resulted in a hospitalization had a mean cost per episode of $5,812 (95%CI 3751–7873) (median $5,271, IQR 4553–8654), including hospitalization costs. These episodes were excluded from further analyses as the focus was on non-hospitalized episodes. The 205 non-hospitalized episodes with complete data had a mean total cost per episode of $241 (95%CI 157–326; median $57, IQR 3–170). After multiple imputation (*n* = 257), the mean total cost per episode was $252 (95%CI 169–334); see Table 2 for costs by sector and timepoint. Given the estimate of total cost per episode using multiple imputation closely approximated the estimate of total cost per episode among the complete cases, the imputed results were used for subsequent analyses.

**Table 2:** Mean (95%CI) cost per episode after multiple imputation, by timepoint, and sector (*n* = 257).

	**Caretakers**	**Health sector**	**Employers**	**Total**
Week 0	6.43 (3.48, 9.37)	48.12 (33.30, 62.94)	11.34 (2.02, 20.65)	65.88 (47.73, 84.04)
Week 1	41.43 (19.13, 63.73)	18.05 (7.69, 28.42)	20.02 (0.00*, 47.36)	79.51 (30.11, 128.91)
Week 2	31.38 (9.57, 53.19)	16.23 (4.71, 27.76)	1.66 (0.00*, 4.09)	49.28 (19.76, 78.80)
Week 3	16.30 (6.64, 25.95)	8.51 (1.52, 15.50)	3.42 (0.00*, 7.62)	28.22 (10.95, 45.49)
Week 4	16.25 (6.08, 26.41)	7.36 (1.96, 12.76)	5.17 (0.00*, 11.45)	28.78 (11.13, 46.43)
Total	111.78 (65.19, 158.38)	98.28 (72.63, 123.93)	41.61 (11.21, 72.01)	251.67 (169.19, 334.16)

* *Negative values for lower 95%CI were manually truncated at zero, given implausibility of negative costs*.

Caretakers incurred the greatest proportion of costs (44%) per episode, followed by the public healthcare system (39%). Employers incurred the lowest proportion of costs (17%; Table 2). Among those episodes with weekly follow-ups, the greatest proportion of costs occurred between Week 0 (time of illness notification) and Week 1 (Table 2). This was consistent with the number of episodes that reported some illness-related resource use at each timepoint (75 at Week 1, 46 at Week 2, 39 at Week 3, and 29 at Week 4). Among those reporting some illness-related resource use, the proportion of parents/guardians who reported not being worried about the amount of money they spent on the cough illness was 5% (*n* = 4/75) at Week 1, 4% (*n* = 2/46) at Week 2, 15% (*n* = 6/39) at Week 3, and 14% (*n* = 4/29) at Week 4.

**Figure 3:**
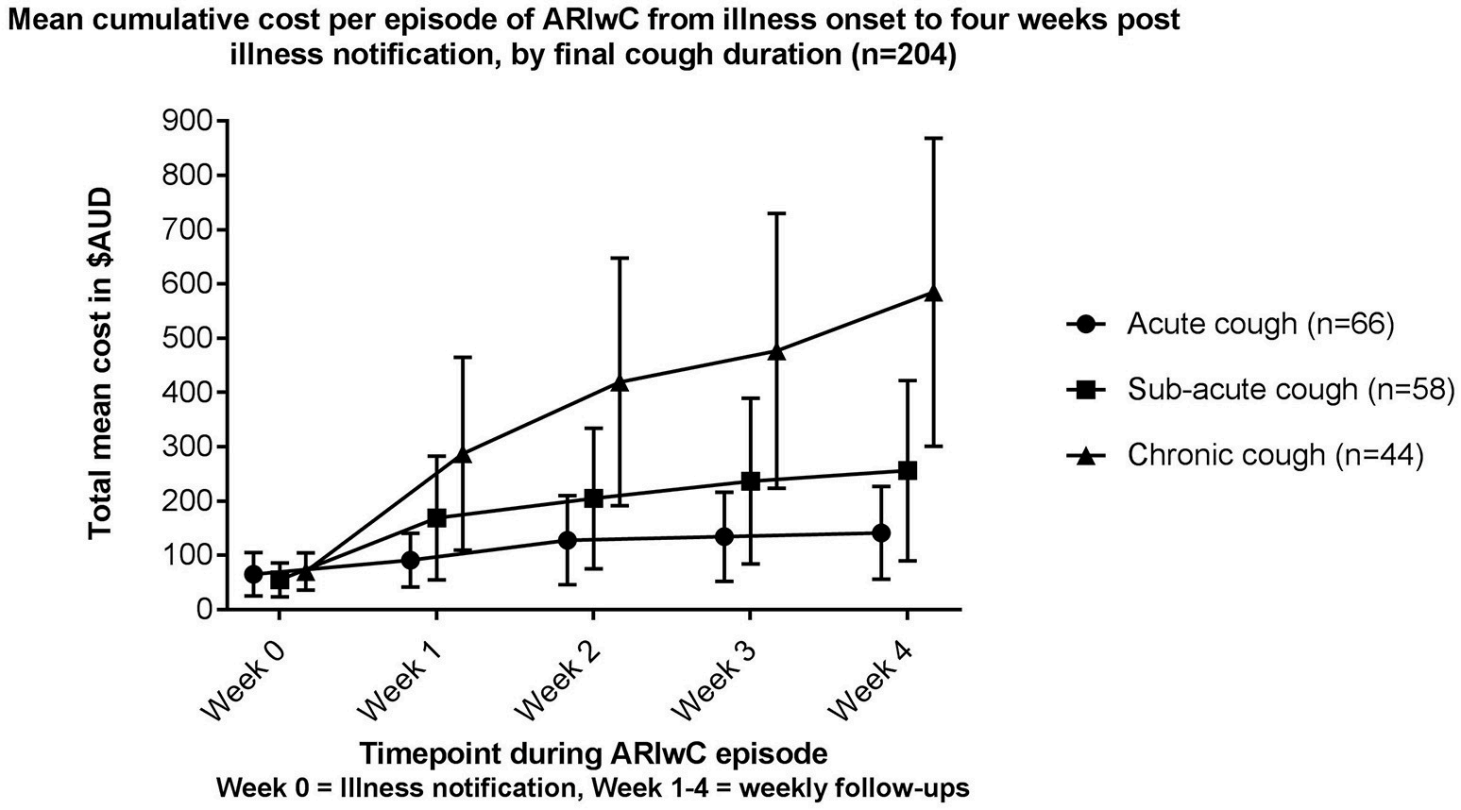
Cumulative cost per episode (means and 95% CI's) of ARIwC from illness onset to 4 weeks post illness notification among episodes requiring weekly follow-ups, by final cough duration (*n* = 204).

There were 86 (33%) episodes in which illness onset was during winter, 77 during autumn (30%), 54 (21%) during summer, and 40 (16%) during spring. The total mean cost of a winter episode was $455 (95%CI 248–661); significantly greater than the total mean cost of spring ($144, 95%CI 37–251, *p* = 0.01), summer ($96, 95%CI 20–172, *p* < 0.01), and autumn ($182, 95%CI 72–291, *p* < 0.01) episodes. There were 107 episodes (42%) classified as acute cough episodes, 66 (26%) as sub-acute, and 44 (17%) as chronic. Among the remaining 40 episodes cough duration was unknown. Chronic cough episodes had a total mean cost per episode of $585 (95%CI 301–868), which was significantly higher than acute cough episodes ($118, 95%CI 61–175, *p* < 0.001) and sub-acute cough episodes ($230, 95%CI 84–377, *p* < 0.01). Figure 3 shows the cumulative cost of illness over the follow-up period among those episodes requiring weekly follow-ups. Among this group, the cost of chronic cough episodes was significantly higher than both acute cough (*p* < 0.01) and sub-acute cough (*p* < 0.05) episodes by Week 2 of the follow-up period.

The total mean cost per child during the study period, unadjusted for months of observation, was $469 (95%CI 301–636). The total cost per child-month of observation due to non-hospitalized ARIwC was $83 (95%CI 43–122). Three baseline characteristics were found to be independently and significantly associated with a higher cost per child-month of observation: (1) having experienced wheezing in the 12 months prior to enrolment (*coeff*. $86, 95%CI 7–165, *p* < 0.05); (2) having connections with traditional lands/homelands (*coeff*. $81, 95%CI 1–160, *p* < 0.05), and; (3) having a parent/guardian who believed that antibiotics prescribed by a doctor should be given until symptoms resolve (*coeff*. $145, 95%CI 11–279, *p* < 0.05).

The adjusted total annual cost was $991 (95%CI 514–1468). The adjusted annual cost was $425 (95%CI 154–697) to caretakers, $426 (95%CI 191–661) to the public healthcare system, and $140 (95%CI 0–288) to employers.

## Discussion

This is the first study to have estimated the costs of non-hospitalized ARIwC episodes among Aboriginal and/or Torres Strait Islander children. The total mean cost of ARIwC was estimated to be $252 per episode, $83 per child month of observation, and $991 per child-year. Approximately half of the cost per episode was incurred by caretakers, one third by the public healthcare system and one fifth by employers. Two episode-related characteristics were associated with a higher cost per episode (season and cough duration) and three child-related characteristics were associated with cost per child month of observation (prior history of wheezing, connection to traditional lands, and parent/guardian understanding of recommended antibiotic duration).

As international studies are not comparable to the Australian context, these findings have been interpreted in the context of other Australian studies. The total mean cost per episode among this cohort was lower than that reported by Lambert et al. ($241 = ~$AU351 after adjusting to 2017 prices from 2001/2002) (12), Lambert et al. ($309 = ~$AU435 after adjusting to 2017 prices from 2003) (13), and Yin et al. ($AU626 = ~$AU724 after adjusting to 2017 prices from 2010) (14). These three studies primarily focused on ILI among children aged <6 years, and two focused exclusively on illnesses during winter and spring (12, 14). The cost differences between this study and those above are likely due to these differences in study characteristics, as well as differences in the socio-economic characteristics of the participating families. Lambert et al. (13) found that children from families in the lowest household income bracket (<$52,000 per year) reported the lowest costs per episode (~$166 less than the cohort mean). Families with an annual household income of <$52,000 made up 24% of their study cohort, compared to 74% of children in this study; 75% of families in Yin et al.'s cohort had an annual household income of $104,000 or more.

Parental employment status and childcare use also differed between our study and Yin et al.'s (14). In the latter, 73% of children had both parents employed, and this characteristic was significantly associated with higher costs per episode of ILI (compared to one parent or no parents) (14). Furthermore, 89% of children in Yin et al.'s cohort were attending childcare compared to 33% in this study. While childcare attendance was not significantly associated with cost in Yin et al. (14), a study conducted in the Netherlands of respiratory illnesses among children aged 0–2 years found the cost per episode of ILI to be twice as much among children attending day care centers than children not attending day care centers (mean cost of €196 compared to €95 in year 2012, respectively) (40). Neither of the Lambert et al. studies (12, 13) described parental employment status or childcare use among their cohorts. However, their cohorts were likely similar in these respects to Yin et al.'s cohort, given similarities in household incomes and the use of childcare centers/playgroups as recruitment settings.

Our cohort's socio-economic characteristics also likely impacted the proportions of costs incurred by each sector. The Lambert studies (12, 13) reported that 87 and 79% of costs per episode of ILI were incurred by the patient and family, respectively (44% in our study) and 5 and 6% of total costs per episode were incurred by the healthcare sector (39% in our study). This is attributable to the availability of Australian government initiatives to assist Aboriginal and/or Torres Strait Islander families to access, utilize and afford health care services, and medications such as bulk-billing (Box 1) and the “Close the Gap” scheme (Box 1). The use of such initiatives amongst this cohort thereby decreased the proportion of costs incurred by caretakers. However, despite these initiatives, the economic burden on caretakers in this study was substantial relative to their capacity to pay. This was evident in that at any timepoint during an ARIwC episode, no more than 20% of parent/guardians reported not being worried about the amount of money spent on their child's cough illness. Being worried about the amount of money spent on a child's ARIwC illness has been reported to negatively impact quality of life (41). This stresses the importance of recognizing the impact of monetary costs on families, as the emotional and social stress of managing an illness in a child may be heightened by the economic burden.

Previous studies have identified an increased risk of young children experiencing an ARI during winter months (3, 7). This study found that the cost of an ARIwC episode with onset in winter was significantly greater than an episode with onset in spring, summer, or autumn. Few other studies have estimated the costs of respiratory infections across all seasons, and those that have (13) have not compared costs between seasons. Lambert et al. (13) examined costs collected over a 12-month period by virus type and found the cost of laboratory confirmed Influenza A (a winter illness in southern Australia) to be higher than episodes with other respiratory viruses.

Approximately one in five ARIwC episodes resulted in chronic cough in this study, similar to previous findings of children presenting to a tertiary pediatric ED with ARIwC (42). The total mean cost of an episode resulting in chronic cough was 2.5 times that of sub-acute cough episodes, and 5.0 times that of acute cough episodes. Few published studies have examined the effect of cough duration on cost per episode. Yin et al. (14) reported greater costs were associated with a longer duration of ILI. However, in the present study episodes that progressed to chronic cough incurred the greatest proportion of costs early in the illness (i.e., during the acute phase). This suggests that it is not the duration of the cough illness itself that is the main cause of higher costs, but rather characteristics of the illness that from the onset affect both the cost and the cough duration. Thus, early identification of children at risk of developing chronic cough and early intervention to prevent the development of chronic cough is important, as the cost savings are likely to be high.

In addition to these two episode-specific characteristics, this study identified three child/family characteristics associated with the cost per child-month of observation. The finding of a prior history of wheeze being associated with higher costs is not unexpected. Perception of a child's vulnerability and susceptibility to severe illness, as a consequence of previous illness, has been reported to be an important influence on parent's decision to seek healthcare (43). A history of wheezing may therefore increase parent/guardian concern, thereby increasing the likelihood of seeking healthcare, and consequently increasing cost of illness. A history of wheezing may also increase the likelihood of the child experiencing wheezing during a current ARI episode and requiring medical attention. Kusel et al. (3) reported that among Australian children aged <5 years with an ARI, the proportion of episodes that incurred a visit to the doctor and/or a hospitalization was greater among episodes with wheeze, than without.

There is little quantitative research exploring the effect that historical and cultural factors have on health and illness among Aboriginal and/or Torres Strait Islander peoples. This present study found that being in a family with connections to traditional lands was associated with higher costs per month of observation. Previous research among this same cohort found that having a family member from the Stolen Generation (Box 1) was associated with having an ARIwC at the time of baseline presentation to the recruitment clinic (44). Indeed these factors are closely related, with 67% of those responding “yes” to connection to traditional lands, also responding “yes” to having a family member from the Stolen Generation. The mechanisms by which cultural and historical factors influence respiratory health and cost of respiratory illness are not well understood, however, their importance is apparent, and consequently further research into this area should be prioritized.

The association between increased cost per month of observation and parent/guardian belief that antibiotics should be given until there are no more symptoms (opposed to until the course is finished) is important. Whether this increase in cost is a result of ARIs not resolving and therefore requiring further medical attention, or whether it reflects a broader issue of health literacy that is influencing the management, and therefore cost, of illness is unclear. However, this finding needs to be interpreted with caution as belief in the required duration of antibiotics may not have been consistent with actual behavior and while the association was significant the number of participants reporting that antibiotics should be given until no more symptoms was small.

A key strength of this study is the almost 4-year period of recruitment and data collection, enabling us to capture costs across all seasons and several years. As none of the other Australian studies (12–14) were >12 months duration, their reported costs may reflect year-specific incidences, etiologies and severities of respiratory infections. However, the generalizability of this study's findings to all Aboriginal and/or Torres Strait Islander children may be somewhat limited by this being a single center study. Nevertheless, 62% of Aboriginal and/or Torres Strait Islander's live in a “Major City” or “Inner Regional” area (45), similar to this community. Further, most major Australian cities have similar healthcare service providers, and Medicare, PBS, and CTG are national schemes. The findings of this study may not be applicable to remote communities, particularly given potential differences in delivery, access and use of health care services as well as the availability of employment and formal childcare services.

A limitation of our study is the missing data; however, multiple imputation was used to account for this. There were some differences in the characteristics of children of parents/guardians who reported ≥1 ARIwC episode during the study period and children of parents/guardians who reported no episodes, particularly with respect to prior respiratory history such as wheezing (Supplementary Table 2). In those children with no ARIwC episodes reported, we are unable to determine whether this was correct or whether it was a reporting bias due to poorer engagement in the study. The latter seems most plausible given children who reported no ARIwC episodes had significantly fewer months of observation than those with ≥1 episode. Another limitation to our study is the 53 episodes which had illness reports but no weekly follow-ups, due to study staff only being notified of the ARIwC illness after illness resolution. The costing of these episodes may have underestimated the cost of illness given the exclusion of “time off non-work activities” in the illness reports and the potential for recall bias. However, these episodes were illnesses with a shorter duration, may also have been less severe, and thus would have likely incurred lower costs per episode regardless.

Our costing methods also had several limitations. Firstly, we did not include transport costs to and from healthcare services, however anecdotally most of our study population were accessing local health services, and/or utilizing public transport or transport services provided by health clinics. Previous studies have reported that transport costs made up <1% (12) of the total cost of illness, so it is unlikely that the exclusion of transport costs would have made a meaningful difference to our findings. Secondly, the unit costs used to estimate the cost of time off work were likely to have been greater than what caretakers were actually being paid, given the low household incomes of most families in this study.

In summary, the cost to caretakers and the public healthcare system to manage and treat ARIwC among children in this population was substantial and was a source of concern for caretakers. While total cost per episode was less than what has been reported in other Australian studies, the proportion of costs incurred per episode by the public healthcare system were greater. The importance of economic considerations in the development and delivery of services, policies and programs to improve the health of Aboriginal and/or Torres Strait Islander peoples has recently been highlighted (46, 47). Our study provides a baseline for which further economic studies in this population can be developed and evaluated. Given differences in costs between episodes occurring in different seasons and cough durations, future research should take this into account, particularly when considering the cost-effectiveness of interventions. While the proportion of parents/guardians who believed that antibiotics should be given until there are no more symptoms was small, when seeking health care parents/guardians may benefit from being reminded of the importance of finishing the prescribed course of antibiotics. The significance of historical and cultural factors on health and well-being cannot be overlooked and future health research among Aboriginal and/or Torres Strait Islander populations should consider examining their influence further.

## Author Contributions

YL-T assisted with data collection, management and cleaning; sourced and applied unit costs; analyzed and interpreted the data; and drafted all versions of the manuscript. SM provided guidance with data analysis. YA-Y assisted with data cleaning and creation of tables and figures. KH was responsible for participant recruitment and data collection. AC conceptualized and designed the overall study. MO provided guidance on study design and sourcing unit costing information. DV provided statistical advice in regards to data analysis. KFO was the chief investigator on the overall study and provided guidance on data analysis and interpretation. All authors were involved in editing versions of the manuscript and appraising the final version.

### Conflict of Interest Statement

The authors declare that the research was conducted in the absence of any commercial or financial relationships that could be construed as a potential conflict of interest.
