# Supplementary material for: The Cost of Acute Respiratory Infections With Cough Among Urban Aboriginal and Torres Strait Islander Children
Source: Front Pediatr. 2018 Dec 3;6:379. doi: 10.3389/fped.2018.00379 (PMC6287573; doi:10.3389/fped.2018.00379)
Supplement: Supplementary file 1 [file Table_1.docx]

Supplementary Material 1

**The cost of acute respiratory infections with cough among urban Aboriginal and Torres Strait Islander children**

**Yolanda G Lovie-Toon^1^*, Steven M McPhail^1,2^, Yin To Au-Yeung^1^, Kerry K Hall^3^, Anne B Chang^1,4,5^, Dimitrios Vagenas^1^, Michael E Otim^6,7^, Kerry-Ann F O’Grady^1^**

*** Correspondence:**

Yolanda Lovie-Toon

[y.lovietoon@qut.edu.au](mailto:y.lovietoon@qut.edu.au)

**Illness Report**

Date of ARI notification: |__|__| |__|__|__| |__|__|__|__|

D D M M M Y Y Y Y

ARI Episode number: |__|__|

Notified by:  Parent/carer  Murri Medical Staff  RiOAR staff

Type of presentation:  Parent reported

Presented to Murri Medical, RiOAR staff not present

Presented to Murri Medical, RiOAR staff present

**Illness details**

Date first symptom started: |__|__| |__|__|__| |__|__|__|__|

D D M M M Y Y Y Y

**Please document symptoms present with this current illness:**

|  | ***Yes*** | ***No*** | ***Unk*** | ***N/A*** | ***# of days symptom***  ***present*** | ***Present today*** | ***Sought medical attention*** |
| --- | --- | --- | --- | --- | --- | --- | --- |
| a. Fever/temp/feel hot |  |  |  |  | \|__\|__\|  Unk | Yes  No  Unk | Yes  No |
| b. Moist/wet cough |  |  |  |  | \|__\|__\|  Unk | Yes  No  Unk | Yes  No |
| c. Dry cough |  |  |  |  | \|__\|__\|  Unk | Yes  No  Unk | Yes  No |
| d. Runny nose |  |  |  |  | \|__\|__\|  Unk | Yes  No  Unk | Yes  No |
| e. Increased tiredness |  |  |  |  | \|__\|__\|  Unk | Yes  No  Unk | Yes  No |
| f. Sore throat |  |  |  |  | \|__\|__\|  Unk | Yes  No  Unk | Yes  No |
| h. Wheeze/whistle |  |  |  |  | \|__\|__\|  Unk | Yes  No  Unk | Yes  No |
| i. Shortness of breath |  |  |  |  | \|__\|__\|  Unk | Yes  No  Unk | Yes  No |
| j. Fast breathing |  |  |  |  | \|__\|__\|  Unk | Yes  No  Unk | Yes  No |
| l. Earache |  |  |  |  | \|__\|__\|  Unk | Yes  No  Unk | Yes  No |
| m. Chills |  |  |  |  | \|__\|__\|  Unk | Yes  No  Unk | Yes  No |
| n. Muscle aches/ pains |  |  |  |  | \|__\|__\|  Unk | Yes  No  Unk | Yes  No |
| o. Headache |  |  |  |  | \|__\|__\|  Unk | Yes  No  Unk | Yes  No |
| p. Irritability |  |  |  |  | \|__\|__\|  Unk | Yes  No  Unk | Yes  No |
| q. Vomiting |  |  |  |  | \|__\|__\|  Unk | Yes  No  Unk | Yes  No |
| r. Diarrhoea***** |  |  |  |  | \|__\|__\|  Unk | Yes  No  Unk | Yes  No |
| t. Unsettled sleep |  |  |  |  | \|__\|__\|  Unk | Yes  No  Unk | Yes  No |
| v. Unable to play |  |  |  |  | \|__\|__\|  Unk | Yes  No  Unk | Yes  No |
| w. Feeding difficulties |  |  |  |  | \|__\|__\|  Unk | Yes  No  Unk | Yes  No |
| x. Poor appetite |  |  |  |  | \|__\|__\|  Unk | Yes  No  Unk | Yes  No |
| y. Toothache |  |  |  |  | \|__\|__\|  Unk | Yes  No  Unk | Yes  No |
| z. Other (specify) |  |  |  |  | \|__\|__\|  Unk | Yes  No  Unk | Yes  No |

*** Note: defined as ≥ 3 loose stools in a 24 hour period**

**I am now going to ask you to score your child’s cough for the past 3 days. Cough score – please let me know which one of these options best describes your child’s cough.**

|  | **Today** | | **Last Night** | **Yesterday** | **1 Night Ago** | **2 Days Ago** | **2 Nights Ago** |
| --- | --- | --- | --- | --- | --- | --- | --- |
| Cough **Score** | \|__\| | | \|__\| | \|__\| | \|__\| | \|__\| | \|__\| |
| **Average** Cough Score | Day Score: \|__\| | | | | Night Score: \|__\| | | |
| **Cough** type | Wet  Dry  Both  No cough  Unknown | Wet  Dry  Both  No cough  Unknown | | Wet  Dry  Both  No cough  Unknown | Wet  Dry  Both  No cough  Unknown | Wet  Dry  Both  No cough  Unknown | Wet  Dry  Both  No cough  Unknown |

| **Day-time cough score (today)** | **Night-time cough score (last night)** |
| --- | --- |
| 0 = No cough during the day | 0 = No cough at night |
| 1 = Cough for one-two short periods only | 1a = Cough on waking only (cough did not wake child up) |
| 2 = Cough for more than two short periods | 1b = Cough on going to sleep only |
| 3 = Frequent coughing but does NOT interfere with school or other daytime activities | 2 = Awoken once or awoken early due to coughing |
| 4 = Frequent coughing which DOES interfere with school or other daytime activities | 3 = Frequent waking due to cough(s) |
| 5 = Cannot perform most usual daytime activity due to severe coughing | 4 = Frequent cough most of the night |
|  | 5 = Distressing cough |

**
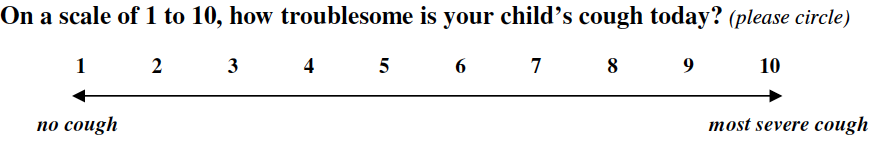
**

**Has the child been seen by any health professionals/services for his/her current illness?**

Yes  No  Unknown

*If yes, tick all that apply:*

Murri Medical # times seen: |__|__|  Unk #days since last seen: |__|__|  Unk

Other AMS GP # times seen: |__|__|  Unk #days since last seen: |__|__|  Unk

Other non-AMS GP # times seen: |__|__|  Unk #days since last seen: |__|__|  Unk

IHW in community # times seen: |__|__|  Unk #days since last seen: |__|__|  Unk

ED/Hospital # times seen: |__|__|  Unk #days since last seen: |__|__|  Unk

Community health nurse # times seen: |__|__|  Unk #days since last seen: |__|__|  Unk

Pharmacist # times seen: |__|__|  Unk #days since last seen: |__|__|  Unk

Natural therapist # times seen: |__|__|  Unk #days since last seen: |__|__|  Unk

Other # times seen: |__|__|  Unk #days since last seen: |__|__|  Unk

(Specify) ________________________________________________

**Did you have to pay for any of those other services above?**  Yes  No  Unk

If yes, which services did you have to pay for? _____________________________________

If yes, how much have you paid so far for these services $|__|__|__|  Unk  Declined

**Was the child hospitalised for this current illness?**  Yes  No  Unk

If yes, name of hospital: __________________________ Admission date: ___/___/___

Number of days in hospital: |__|__|

**Have any investigations been performed for this current illness?**  Yes  No  Unk

If yes, complete table below

| Type | Who ordered test? | Has test been done? | Cost to patient? | Results to chase? |
| --- | --- | --- | --- | --- |
| Blood test |  |  |  |  |
| Chest xray |  |  |  |  |
| Nose swab |  |  |  |  |
| Throat swab |  |  |  |  |
| Other |  |  |  |  |

Has the child been given any medications for this illness?  Yes  No  Unk

*If yes, complete the following*

If yes, complete the following:

| **Name** | **Dose per day** | **Date started** | **Number of days** | **Reason** |
| --- | --- | --- | --- | --- |
| Unk | Unk | Unk | Unk | Unk |
| Unk | Unk | Unk | Unk | Unk |
| Unk | Unk | Unk | Unk | Unk |
| Unk | Unk | Unk | Unk | Unk |

Has the child’s primary carer had to take time of work

because of this illness?  Yes  No  Unk  N/A

*If yes, how many days off work?* |__|__|

Has anyone else had to take time of work because of this illness?  Yes  No  Unk  N/A

*If yes, how many days off work?* |__|__|

How many people have been involved in helping care for your child

while he/she has been sick? |__|__|  Unk

*Specify who has helped care for the child other than primary carer:*

Partner/spouse  Aunty  Uncle  Grandparent  Sibling  Friend  Other

If the child goes to childcare/kindy, has the child had time off

childcare/kindy?  Yes  No  Unk  N/A

*If yes, how many days off child-care/kindy?* |__|__|  Unk

Have any other commitments you have had to be cancelled

because of this illness?  Yes  No  Unk  N/A

*If yes, specify_________________________________________________________________________*

C1. Has an anterior nasal swab been collected as per protocol?

Yes, both nares

No, one nare only

No specimens collected

*C1a Reason only one nare or none collected:__________________________________________________*

C2. Who collected the specimen?  Research staff  Murri Medical Staff  Carer

C2. Time specimen collected: ____:_____hrs

**Specimen ID number: LSIMKS** |__|__|__|__|

***C3 Record quality of specimen collection technique***

|  | Left nare | Right nare |
| --- | --- | --- |
| Good (ie tip inserted 1cm , turned 4 times against internal nare) |  |  |
| Fair (tip partially inserted or complete swabbing (4 turns) not achieved |  |  |
| Poor (difficulty in collection and unlikely to have obtained good/fair sample) |  |  |
| Not done |  |  |
| Not applicable (NPA sample collected instead) |  |  |

C3a Comments on specimen collection: __________________________________________________

___________________________________________________________________________________

***SPECIMEN CHECK LIST***

Specimen labelled correctly

Specimen placed in biohazard bag and refrigerated

**Medical Diagnosis**

If the child was seen by a doctor, specify the doctor’s primary diagnosis:

______________________________________________________________________

Remind carer of next follow-up in one week’s time

Ensure carer has enough cough diary cards

Name of person completing follow-up: ____________________________________________

Signature: _________________________________

Date: |__|__| |__|__|__| |__|__|__|__|

Comments

**____________________________________________________________________________**

**____________________________________________________________________________**

**____________________________________________________________________________**

**____________________________________________________________________________**

**____________________________________________________________________________**

**____________________________________________________________________________**

**____________________________________________________________________________**

**____________________________________________________________________________**

**____________________________________________________________________________**


**Weekly Follow-Ups (repeated at day 14, day 21 and day 28 post illness-notification)**

**Respiratory illness: Day 7 Follow-up (Specimen collection)**

**Contact attempt 1**

Date: ___/___/___ Time: ___:___hrs Contact method:  Phone  SMS  Email

Contact successful (ie parent responds to message/call):  Yes  No

**Contact attempt 2**

Date: ___/___/___ Time: ___:___hrs Contact method:  Phone  SMS  Email

Contact successful (ie parent responds to message/call):  Ye s  No

**Contact attempt 3**

Date: ___/___/___ Time: ___:___hrs Contact method:  Phone  SMS  Email

Contact successful (ie parent responds to message/call):  Yes  No

Person completing interview :  Primary carer  Other (specify) ________________________________________

1. Does the child have a cough today?  Yes  No  Unknown

2. If yes, what type of cough is it?  Wet  Dry  Both  Unknown

1. **What is your child’s cough score for today and for last night (circle appropriate score?**

| **Day-time cough score (today)** | **Night-time cough score (last night)** |
| --- | --- |
| 0 = No cough during the day | 0 = No cough at night |
| 1 = Cough for one-two short periods only | 1a = Cough on waking only (cough did not wake child up) |
| 2 = Cough for more than two short periods | 1b = Cough on going to sleep only |
| 3 = Frequent coughing but does NOT interfere with school or other daytime activities | 2 = Awoken once or awoken early due to coughing |
| 4 = Frequent coughing which DOES interfere with school or other daytime activities | 3 = Frequent waking due to cough(s) |
| 5 = Cannot perform most usual daytime activity due to severe coughing | 4 = Frequent cough most of the night |
|  | 5 = Distressing cough |
|  |  |

1. Has your child stopped coughing for 3 or more days in the past week?  Yes  No  Unk
2. **In the past week has your child had any medications for their cough illness?**  Yes  No  Unk

| **Name** | **Number of days** | **Doses/day** | **Indication?** |
| --- | --- | --- | --- |
| ________________ | ____________ | _____________ | _____________________ |
| ________________ | ____________ | _____________ | _____________________ |
| ________________ | ____________ | _____________ | _____________________ |
| ________________ | ____________ | _____________ | _____________________ |

1. **In the past week did your child see a local doctor (GP) for their cough illness?**  Yes  No  Unknown
2. If yes, number of times? |__|__| times
3. Was the appointment(s) bulk billed?  Yes  No  Unknown
4. Which GP did you go to?  Murri Medical  Other AMS  Mainstream GP
5. **In the past week did your child go to an emergency department for their cough?**  Yes  No  Unknown
6. If yes, number of times? |__|__| times
7. Was your child admitted to hospital?  Yes  No  Unknown
8. If yes, how many days was the child in hospital? |__|__| days
9. Was your child admitted using private health insurance?  Yes  No  Unk

**In the past week did your child see any other healthcare providers for their cough illness?**

1. **If yes, what sort?**

| **Type** | **Number of visits** | **Payed for by family?** |
| --- | --- | --- |
| ______________________ | ____________ | Yes  No |
| ______________________ | ____________ | Yes  No |
| ______________________ | ____________ | Yes  No |
| ______________________ | ____________ | Yes  No |

1. **In the past week has your child had any tests done for their cough illness?**  Yes  No  Unk
2. **If yes, what type:**

| **Type of Test** | **Number performed** | **Payed for by family?** |
| --- | --- | --- |
| ______________________ | _________________ | Yes  No |
| ______________________ | _________________ | Yes  No |
| ______________________ | _________________ | Yes  No |
| ______________________ | _________________ | Yes  No |

1. **If yes to the above healthcare questions, did you attend the appointment(s) with the child?**Yes  No  NA
   1. Total time spent seeking healthcare: _____hours _____minutes
   2. How much of the time spent seeking healthcare was:
      1. Time off work with pay lost? _____hours _____minutes
      2. Time off work with no pay lost? _____hours _____minutes
      3. Time off usual activity (not work)? _____hours _____minutes
2. **Did anyone else attend these appointments with your child?**  Yes  No  NA

**Person 1**

- 1. Relationship to child: _______________ Gender:  Male  Female

1. Total time spent seeking healthcare: _____hours _____minutes
2. How much of the time spent seeking healthcare was:
   - 1. Time off work with pay lost? _____hours _____minutes
     2. Time off work with no pay lost? _____hours _____minutes
     3. Time off usual activity (not work)? _____hours _____minutes

**Person 2**

- 1. Relationship to child: _______________ Gender:  Male  Female

1. Total time spent seeking healthcare: _____hours _____minutes
2. How much of the time spent seeking healthcare was:
3. Time off work with pay lost? _____hours _____minutes
4. Time off work with no pay lost? _____hours _____minutes
5. Time off usual activity (not work)? _____hours _____minutes
6. **Other than time spent seeking healthcare for your child’s cough, did you spend extra time (time in excess of that normally spent caring for the child) caring for your child in the past week?**  Yes  No
   1. Total time spent caring for child: _____hours _____minutes
   2. How much of the time spent seeking healthcare was:
      1. Time off work with pay lost? _____hours _____minutes
      2. Time off work with no pay lost? _____hours _____minutes
      3. Time off usual activity (not work)? _____hours _____minutes
7. **Other than time spent seeking healthcare for your child’s cough, did anyone else spend extra time (time in excess of that normally spent caring for the child) caring for your child in the past week?**  Yes  No

**Person 1**

- 1. Relationship to child: _______________ Gender:  Male  Female
  2. Total time spent caring for child: _____hours _____minutes
  3. How much of the time spent seeking healthcare was:
     1. Time off work with pay lost? _____hours _____minutes
     2. Time off work with no pay lost? _____hours _____minutes
     3. Time off usual activity (not work)? _____hours _____minutes

**Person 2**

1. Relationship to child: _______________ Gender:  Male  Female
2. Total time spent caring for child: _____hours _____minutes
3. How much of the time spent seeking healthcare was:
   - 1. Time off work with pay lost? _____hours _____minutes
     2. Time off work with no pay lost? _____hours _____minutes
     3. Time off usual activity (not work)? _____hours _____minutes
4. **In the past week did your child stay away from arranged childcare because of their cough?** Yes  No  NA
   1. If yes, was money lost due to fees already paid or requiring payment?  Yes  No
   2. If yes, how much money was lost? $___________
5. **In the past week did your child miss any planned activities because of their cough illness?**  Yes  No
   1. If yes, was money lost due to fees already paid or requiring payment?  Yes  No
   2. If yes, how much money was lost? $___________
6. **In the past week did you miss any planned activities because of your child’s cough illness?**  Yes  No
   1. If yes, was money lost due to fees already paid or requiring payment?  Yes  No
   2. If yes, how much money was lost? $___________
7. **In the past week did anyone else miss any planned activities because of your child’s cough?**  Yes  No
   1. If yes, was money lost due to fees already paid or requiring payment?  Yes  No
   2. If yes, how much money was lost? $___________
8. **Have you incurred any other costs because of your child’s cough illness not already covered?**  Yes  No
   1. **If yes,**

| **What for?** | **Cost?** |
| --- | --- |
| ________________________________ | $_______________ |
| ________________________________ | $_______________ |
| ________________________________ | $_______________ |

**18. Since your last appointment how worried/concerned were you about the amount of money lost due to your child’s cough illness?**

Very, Very worried/concerned Very worried/concerned Fairly worried/concerned

Somewhat worried/concerned A little worried/concerned Hardly worried/concerned Not worried/concerned

Remind parent to continue daily diary card

**Remind parent to collect nose swab and put in fridge**

Parent reminded of next follow-up contact at day 14 (± 3 days) and need to take nose swab on that day

RA name:________________________________Signature: _________________________________

Date: |__|__| |__|__|__| |__|__|__|__|
