# Supplementary material for: The Cost of Acute Respiratory Infections With Cough Among Urban Aboriginal and Torres Strait Islander Children
Source: Front Pediatr. 2018 Dec 3;6:379. doi: 10.3389/fped.2018.00379 (PMC6287573; doi:10.3389/fped.2018.00379)
Supplement: Supplementary file 2 [file Table_2.docx]

Supplementary Material

The cost of acute respiratory infections with cough among urban Aboriginal and Torres Strait Islander children

Yolanda G Lovie-Toon^1^*, Steven M McPhail^1,2^, Yin To Au-Yeung^1^, Kerry K Hall^3^, Anne B Chang^1,4,5^, Dimitrios Vagenas^1^, Michael E Otim^6,7^, Kerry-Ann F O’Grady^1^

*** Correspondence:**

Yolanda Lovie-Toon

[y.lovietoon@qut.edu.au](mailto:y.lovietoon@qut.edu.au)

| Supplementary Table 2: Self-reported baseline characteristics of children eligible for inclusion in analysis, of children who reported at least one ARIwC illness during the study period and of children who reported no ARIwC illnesses during the study period | | | | |
| --- | --- | --- | --- | --- |
|  | All children,  *n=178, n (%)* | Children with ≥1 ARIwC illness, *n=138, n (%)* | Children with 0 ARIwC illnesses, *n=40, n (%)* | p-value |
| DEMOGRAPHIC CHARACTERISTICS | | | | |
| Child’s sex | | | | |
| Male  Female | 91 (51.12)  87 (48.88) | 67 (48.55)  71 (51.45) | 24 (60.00)  16 (40.00) | 0.202 |
| Mum’s age (years) | | | | |
| ≤19  20-24  25-29  30-34  35+  Unknown^2^ | 30 (16.85)  62 (34.83)  36 (20.22)  29 (16.29)  20 (11.24)  1 (0.56) | 18 (13.04)  52 (37.68)  29 (21.01)  20 (14.49)  19 (13.77)  0 (0.00) | (30.00)  10 (25.00)  7 (17.50)  9 (22.50)  1 (2.50)  1 (2.50) | 0.016 |
| Dad’s age (years) | | | | |
| ≤19  20-24  25-29  30-34  35+  Unknown^2^ | 8 (4.49)  56 (31.46)  46 (25.84)  22 (12.36)  45 (25.28)  1 (0.56) | 7 (5.07)  45 (32.61)  35 (25.36)  14 (10.14)  37 (26.81)  0 (0.00) | 1 (2.50)  11 (27.50)  11 (27.50)  8 (20.00)  8 (20.00)  1 (2.50) | 0.464 |
| Mother’s highest level of education | | | | |
| Did not finish high school  Finished high school  Higher levels  Unknown/declined/ missing^2^ | 67 (37.64)  84 (47.19)  25 (14.04)  2 (1.12) | 47 (34.06)  70 (50.72)  21 (15.22)  0 (0.00) | 20 (50.00)  14 (35.00)  4 (10.00)  2 (5.00) | 0.118 |
| Father’s highest level of education | | | | |
| Did not finish high school  Finished high school  Higher levels  Unknown/declined/ missing | 80 (44.94)  51 (28.65)  22 (12.36)  25 (14.04) | 58 (42.03)  46 (33.33)  18 (13.04)  16 (11.59) | 22 (55.00)  5 (12.50)  4 (10.00)  9 (22.50) | 0.025 |
| Mother’s employment status | | | | |
| Employed  Unemployed  Unknown^2^ | 25 (14.04)  152 (85.39)  1 (0.56) | 22 (15.94)  116 (84.06)  0 (0.00) | 3 (7.50)  36 (90.00)  1 (2.50) | 0.297^1^ |
| Father’s employment status | | | | |
| Employed  Unemployed  Unknown/ declined | 77 (43.26)  75 (42.13)  26 (14.61) | 59 (42.75)  62 (44.93)  17 (12.32) | 18 (45.00)  13 (32.50)  9 (22.50) | 0.199 |
| Total annual household income ($AUD) | | | | |
| <26,000  26000 - <52000  52000 and above | 68 (38.20)  68 (38.20)  42 (23.60) | 46 (33.33)  56 (40.58)  36 (25.09) | 22 (55.00)  12 (30.00)  6 (15.00) | 0.044 |
| Primary carer on government benefits | | | | |
| Yes  No | 160 (89.89)  18 (10.11) | 124 (89.86)  14 (10.14) | 36 (90.00)  4 (10.00) | 1.000^1^ |
| Family has private insurance that covers the child | | | | |
| Yes  No | 9 (5.06)  169 (94.94) | 7 (5.07)  131 (94.93) | 2 (5.00)  38 (95.00) | 1.000^1^ |
| HOUSEHOLD CHARACTERISTICS | | | | |
| Care type at home | | | | |
| Both parents at home  Single Parent  Other | 99 (55.62)  68 (38.20)  11 (6.18) | 81 (58.70)  49 (35.51)  8 (5.80) | 18 (45.00)  19 (47.50)  3 (7.50) | 0.309 |
| Child’s primary carer | | | | |
| Mother  Other | 165 (92.70)  13 (7.30) | 128 (92.75)  10 (7.25) | 37 (92.50)  3 (7.50) | 1.000^1^ |
| Child’s primary carer identifies as Aboriginal and/or Torres Strait Islander | | | | |
| Yes | 128 (71.91) | 99 (71.74) | 29 (72.50) | 0.925 |
| No | 50 (28.09) | 39 (28.26) | 11 (27.50) |  |
| Number of children living in the household | | | | |
| 0  1  2  3+  Missing^2^ | 38 (21.35)  53 (29.78)  45 (25.28)  41 (23.03)  1 (0.56) | 31 (22.46)  37 (26.81)  37 (26.81)  33 (23.91)  0 (0.00) | 7 (17.50)  16 (40.00)  8 (20.00)  8 (20.00)  1 (2.50) | 0.414 |
| Number of people usually living in the household | | | | |
| 2 - 3  4 - 5  6+  Unknown/declined^2^ | 54 (30.34)  78 (43.82)  45 (25.28)  1 (0.56) | 41 (29.71)  61 (44.20)  36 (26.09)  0 (0.00) | 13 (32.50)  17 (42.50)  9 (22.50)  1 (2.50) | 0.886 |
| Condition of the house in which the child usually lives | | | | |
| Excellent  Good  Average  Poor/Very poor  Missing^2^ | 31 (17.42)  96 (53.93)  33 (18.54)  17 (9.55)  1 (0.56) | 25 (18.12)  75 (54.35)  23 (16.67)  15 (10.87)  0 (0.00) | 6 (15.00)  21 (52.50)  10 (25.00)  2 (5.00)  1(2.50) | 0.465 |
| Mold, mildew, dampness and/or smell in house in which child usually lives | | | | |
| Yes  No  Unknown/declined^2^ | 61 (34.27)  116 (65.17)  1 (0.56) | 47 (34.06)  91 (65.94)  0 (0.00) | 14 (35.00)  25 (62.50)  1 (2.50) | 0.831 |
| CHILD AND FAMILY MEDICAL HISTORY CHARACTERISTICS | | | | |
| Child ever had a cough lasting > 4 weeks | | | | |
| Yes  No  Missing^2^ | 43 (24.16)  134 (75.28)  1 (0.56) | 37 (26.81)  101 (73.19)  0 (0.00) | 6 (15.00)  33 (82.50)  1 (2.50) | 0.142 |
| Child diagnosed with a respiratory illness by a doctor in 12 months prior to enrolment | | | | |
| Yes  No  Missing^2^ | 60 (33.71)  117 (65.73)  1 (0.56) | 52 (37.68)  86 (62.32)  0 (0.00) | 8 (20.00)  31 (77.50)  1 (2.50) | 0.055^1^ |
| Child hospitalized for an acute respiratory illness in 12 months prior to enrolment | | | | |
| Yes  No  Unknown/declined^2^ | 20 (11.24)  157 (88.20)  1 (0.56) | 17 (12.32)  121 (87.68)  0 (0.00) | 3 (7.50)  36 (90.00)  1 (2.50) | 0.571^1^ |
| Child had wheezing in 12 months prior to enrolment | | | | |
| Yes  No  Missing^2^ | 65 (36.52)  112 (62.92)  1 (0.56) | 57 (41.30)  81 (58.70)  0 (0.00) | 8 (20.00)  31 (77.50)  1 (2.50) | 0.017^1^ |
| Child has eczema, currently or previously | | | | |
| Yes  No | 23 (12.92)  155 (87.08) | 21 (15.22)  117 (84.78) | 2 (5.00)  38 (95.00) | 0.111^1^ |
| Family history of reactive airways disease | | | | |
| Yes  No  Unknown/missing^2^ | 132 (74.16)  42 (23.60)  4 (2.25) | 106 (76.81)  30 (21.74)  2 (1.45) | 26 (65.00)  12 (30.00)  2 (5.00) | 0.225 |
| Family history of other chronic lung diseases | | | | |
| Yes  No  Unknown/missing^2^ | 44 (24.72)  130 (73.03)  4 (2.25) | 37 (26.81)  99 (71.74)  2 (1.45) | 7 (17.50)  31 (77.50)  2 (5.00) | 0.271 |
| CULTURAL CHARACTERISTICS | | | | |
| Family maintains cultural connections at home | | | | |
| Yes  No  Unknown^2^ | 110 (61.80)  66 (37.08)  2 (1.12) | 89 (64.49)  49 (35.51)  0 (0.00) | 21 (52.50)  17 (42.50)  2 (5.00) | 0.298 |
| Family has a connection with traditional lands/homelands | | | | |
| Yes  No  Unknown^2^ | 78 (43.82)  91 (51.12)  9 (5.06) | 64 (46.38)  70 (50.72)  4 (2.90) | 14 (35.00)  21 (52.50)  5 (12.50) | 0.412 |
| Child spent time in an Aboriginal/Torres Strait Islander community outside Brisbane in 12 months prior to enrolment | | | | |
| Yes  No  Unknown^2^ | 21 (11.80)  156 (87.64)  1 (0.56) | 19 (13.77)  119 (86.23)  0 (0.00) | 2 (5.00)  37 (92.50)  1 (2.50) | 0.171^1^ |
| Child has any family members from the Stolen Generation | | | | |
| Yes  No  Unknown/declined^2^ | 82 (46.07)  41 (23.03)  55 (30.90) | 69 (50.00)  34 (24.64)  35 (25.36) | 13 (32.50)  7 (17.50)  20 (50.00) | 0.012 |
| RISK FACTOR CHARACTERISTICS | | | | |
| Child’s gestational age (weeks) | | | | |
| < 37  ≥ 37 | 19 (10.67)  159 (89.33) | 15 (10.87)  123 (89.13) | 4 (10.00)  36 (90.00) | 1.000 |
| Child’s birthweight (grams) |  |  |  |  |
| <2500  ≥2500 | 30 (16.85)  148 (83.15) | 26 (18.84)  112 (81.16) | 4 (10.00)  36 (90.00) | 0.235^1^ |
| Child admitted to neonatal intensive care unit for breathing issues post-birth | | | | |
| Yes  No  Unknown/declined^2^ | 22 (12.36)  154 (86.52)  2 (1.12) | 18 (13.04)  120 (86.96)  0 (0.00) | 4 (10.00)  34 (85.00)  2 (5.00) | 0.788^1^ |
| Mother smoked during pregnancy | | | | |
| Yes  No  Unknown/declined^2^ | 87 (48.88)  90 (50.56)  1 (0.56) | 68 (49.28)  70 (50.72)  0 (0.00) | 19 (47.50)  20 (50.00)  1 (2.50) | 0.951 |
| Mother exposed to household smoke during pregnancy | | | | |
| Yes  No  Missing^2^ | 119 (66.85)  58 (32.58)  1 (0.56) | 92 (66.67)  46 (33.33)  0 (0.00) | 27 (67.50)  12 (30.00)  1 (2.50) | 0.763 |
| Child currently exposed to smoke in the household or car | | | | |
| Yes  No  Unknown/declined^2^ | 127 (71.35)  50 (28.09)  1 (0.56) | 99 (71.74)  39 (28.26)  0 (0.00) | 28 (70.00)  11 (27.50)  1 (2.50) | 0.995 |
| Child was, or currently is, breastfed | | | | |
| Yes  No  Missing^2^ | 129 (72.47)  48 (26.97)  1 (0.56) | 104 (75.36)  34 (24.64)  0 (0.00) | 25 (62.50)  14 (35.00)  1 (2.50) | 0.163 |
| Up to date with immunisations with respect to age at enrolment and as per Qld Immunisation Schedules | | | | |
| Yes  No  N/A (aged <3mths)  Missing | 43 (24.16)  116 (65.17)  18 (10.11)  1 (0.56) | 33 (23.91)  90 (65.22)  15 (10.87)  0 (0.00) | 10 (25.00)  26 (65.00)  3 (7.50)  1 (2.50) | 0.412^1^ |
| Child currently attends preschool or childcare | | | | |
| Yes  No  Unknown/declined^2^ | 58 (32.58)  119 (66.85)  1 (0.56) | 46 (33.33)  92 (66.67)  0 (0.00) | 12 (30.00)  27 (67.50)  1 (2.50) | 0.763 |
| PARENT/GUARDIAN COUGH KNOWLEDGE | | | | |
| Is a cough that goes on for more than 4 weeks normal or abnormal? | | | | |
| Normal  Abnormal  Question not asked^3^ | 8 (4.49)  124 (69.66)  46 (25.84) | 8 (5.80)  95 (68.84)  35 (25.36) | 0 (0.00)  29 (72.50)  11 (27.50) | 0.367^1^ |
| Is a wet sounding cough normal or abnormal? | | | | |
| Normal  Abnormal  Unknown^2^  Question not asked^3^ | 6 (3.37)  124 (69.66)  2 (1.12)  46 (25.84) | 4 (2.90)  97 (70.29)  2 (1.45)  35 (25.36) | 2 (5.00)  27 (67.50)  0 (0.00)  11 (27.50) | 0.730^1^ |
| Do you think your child should get antibiotics when he/she has a cold or the flu? | | | | |
| Yes  No  Unknown  Question not asked^3^ | 46 (25.84)  56 (31.46)  30 (16.85)  46 (25.84) | 34 (24.64)  49 (35.51)  20 (14.49)  35 (25.36) | 12 (30.00)  7 (17.50)  10 (25.00)  11 (27.50) | 0.131 |
| If your child is prescribed antibiotics by your doctor, how long should you give them to your child for? | | | | |
| Until no more symptoms  Until they are all finished  Unknown^2^  Question not asked^3^ | 17 (9.55)  113 (63.48)  2 (1.12)  46 (25.84) | 12 (8.70)  91 (65.84)  0 (0.00)  35 (25.36) | 5 (12.50)  22 (55.00)  2 (5.00)  11 (27.50) | 0.588 |

^1^Fisher’s exact test used instead of chi-square because ≥1 cell has cell count <5

^2^Category excluded from analysis of differences between groups because <5 in the unknown group

^3^Question not asked because cough knowledge questions were not added to the baseline questionnaire until part-way through recruitment
